# Diseases of the Circulation

**Published:** 1906-03-03

**Authors:** 


### DISEASES OF THE CIRCULATION.

**Angina Pectoris.** — Thomas Oliver[1] discusses the varieties and the theories as to the causation of this affection. There is (1) the type due to organic lesions, such as aortitis, degeneration, coronary disease, and valvular troubles; (2) the toxic type from thyroid disease, excess in tobacco, or high living; (3) the neurosal; (4) reflex; and (5) vaso-motor types; (6) the forms where angina exists without pain, and pain without angina, which are perhaps more often associated with other kinds of heart disease. This division may be contrasted with Douglas Powell's, who gives (*a*) vaso-motor angina, a neurosis with spasmodic contraction of the systemic, pulmonary, or coronary vessels; (*b*) angina gravior of a primary type due to degenerative or valvular disease of the heart alone; (*c*) secondary cardiac angina, where the spasmodic vaso-contraction is combined with organic heart lesions. Both writers seem to allow that ordinary organic lesions are sometimes accompanied with anginal symptoms, both accept a vaso-motor type; but Oliver adds other groups, such as the neurosal one. As to the causation Oliver notices the difficulties in accepting the theories which refer the affection to (1) spasm of the coronary arteries; (2) spasm of the muscle fibres; (3) spasm of the muscle due to blocking of the coronary arteries; (4) neuritis of the heart or vessels, and in pseudo-angina neuritis of the intercostal and other nerves. He however points out that some lesion of the heart or its vessels has been found in every case of angina gravior, and that not merely a recent one. Still it is uncertain whether functional angina never merges into and becomes true angina, or, indeed, whether a neuralgia itself of sufficient intensity may not kill. Francis Hare,[2] recognising the difficulty in supposing that vaso-constriction of the coronary arteries can occur, suggests that the pain may be due to vascular distension in the entire mediastinum which is compensatory to peripheral constriction and dependent on the activity of the ventricle and the integrity of the mitral valve. If either of these factors are absent the spasmodic attacks cease to occur.

**Bradycardia.**—In an interesting case referred to last year in these columns it was argued that one factor in certain cases of bradycardia is an extraordinary high tension against which a feeble heart struggles to maintain the circulation. Another peculiarity in a case reported by John Hay[3] consisted in the auricular contractions being two or three times as frequent as those of the ventricle, showing that a block occurring in the transmission of the wave of contraction at the sinus. The case was one of paroxysmal type, with a pulse which was slow even during the intervals, and the heart was greatly hypertrophied and dilated. Hay points out that if the ventricle is cut off from the auricular wave, it originates a slow, independent rhythm of its own, and in this case marked pathologic changes were found at the junction. Simultaneous tracings taken from the jugular pulse and the carotid showed that the interval between the contractions of the auricles and ventricles was double the normal time. An interesting question arises as to the extra sounds heard in some of these cases between the ventricular contractions which some observers have ascribed to muscular contractions of the auricles, and others to the auricle forcing blood into the partially filled ventricle, and others, again, to abortive ventricular contractions which do not affect the radial pulse. Hay prefers the second theory, and remarks that an abortive ventricular contraction could hardly be followed immediately by a strong full one, for sometimes one of these numerous weak pulsations closely precedes the ventricular pulsation. An important paper on the subject of bradycardia by G. A. Gibson was published in the *Edinburgh Medical Journal* of July last. He agrees with Hay and Mackenzie that the cases where the jugular pulsations are more numerous than the radial ones are due to a block in the conduction of the wave of contraction; while in cases where the heart is slowed down as a whole, there is either deficient stimulation or a lessened excitability of the heart muscle. Finally there are instances of irregular pulsation in every part of the heart. In this last case there is no loss of conductivity, but the ventricular systole, like the auricular, frequently fails to send on a wave. Here, too, the ventricular systole may be quite independent of the auricular one. Erlanger's observation is worth remembering that the auricular beat can be influenced by atropine, while the ventricular can not. Therefore, when there is a "block" in the conduction of the wave, atropine is useless. Greiwe reports another case of bradycardia, where he found aortic valvular disease and marked changes in the coronary arteries. He thinks that the interference with the blood supply of the heart as a whole led to a damage of the muscle, which increased the "refractory period" of that muscle—namely, prolonged the diastole. However, Stengel remarks that the disease can be produced without any apparent lesion of the heart. In a series collected by Ede, five out of 31 cases showed no sclerotic or muscular changes; but if we accept the view that many of these cases are due to a block in conduction, an almost imperceptible lesion in the auriculo ventricular "bundle of His" would be sufficient. Physiologists have shown that the wave is conducted, not by nerves, but from muscle fibre to muscle fibre, the septum being bridged over by this tiny bundle. If it is cut through or clamped the auricles and ventricles cease to beat at the same rate, and the clinical picture of bradycardia of the Stokes-Adams type is produced. Stengel reports an instance where the bundle of His alone was actually involved by a patch of sclerotic endocarditis, and typical slow pulse with seizures and rapid pulsation of veins followed. The pulse-rate at times was only 16 to 20, while the venous pulsation in the neck was 80 to 100. The syncopal seizures became very frequent, sometimes occurring every half-hour. Before and during these attacks the radial pulse would cease for periods varying from 20 seconds to two minutes and ten seconds, but the venous pulsation went on at its rapid rate during the whole time. Thus there was a condition of complete heart block, as shown by the entire absence of any connection between the auricular and ventricular contractions. The blood-pressure did not appear to be more than 135 during the systole. When an autopsy was made the heart was found unaffected by any degenerative change except in the middle portion of the auriculo-ventricular bundle. The coronary vessels were neither calcareous nor narrowed.

**Choristers' Heart.**—Macnaughton[6] raises the question whether early training in singing and voice production does not tend to cause enlargement of the heart, and especially of its right side, together with a proneness to palpitation, various pains, and mental depression, such as follow overstrain in athletes. He finds in many boys who are undergoing choir training left-sided enlargement of the chest, with a fulness of the veins of the upper extremity, pulsation visible over an abnormally large area, together with shortness of breath and palpitation. These conditions tend to become permanent. In singing, one deep inspiration is made to suffice during a long period, and the pulmonary vessels are kept engorged during that time. As the abdominal muscles are held tense also, the heart becomes distended with blood, and thus becomes dilated and hypertrophied. He bases his theory on observations on a number of cases, in none of whom the heart changes could be referred to rheumatism or other ordinary causes.

[1] Lancet, Sept. 16. [2] Ibid., Sept. 31. [3] Brit. Med. Jour., Oct. 21. [4] Med. Chron., Oct. [5] Amer. Jour. Med. Sci., Dec. [6] Lancet, Oct. 14.

